# Observations on Certain Horny Excrescences of the Human Body

**Published:** 1792

**Authors:** Everard Home


XII. *Obſervations on certain horny Excreſcences of the human Body. By* Everard Home, *Eſq. F. R. S.*—Vide *Philoſophical Tranſactions of the Royal Society of London, Vol.* LXXXI. *for the Year* 1791. *Part* I. 4to. London, 1791.

WE have here an account of a diſeaſe, very remarkable in its effects, but very little underſtood as to its cauſe, namely, the production of an excreſcence ſimilar to horn. Excreſcences of this ſort, ariſing from the human body, have ſometimes been met with in this and other countries; and the horns themſelves have been depoſited as valuable curioſities in different collections in Europe.

Mr. Home obſerves, that, in giving the hiſtory of a diſeaſe ſo rare in its occurrence, and in its effects ſo remarkable as almoſt to exceed belief, it might be thought right to take ſome pains in bringing proofs to aſcertain that ſuch a diſeaſe does really exiſt; but he conſiders the doing ſo as leſs neceſſary at preſent, there being two women now alive, and reſiding in England, who are affected by this complaint. Of theſe two caſes we have here a very full and diſtinct account, account, which ſhows the progreſs of the diſeaſe through its different ſtages.

The ſubject of the firſt of theſe caſes is Mrs. Lonſdale, fifty-ſix years old, and a native of Horncaſtle in Lincolnſhire, who, fourteen years ago, obſerved a moveable tumor on the left ſide of her head, about two inches above the upper arch of the left ear, which gradually increaſed in the courſe of four or five years to the ſize of a pullet's egg, when it burſt, and for a week continued to diſcharge a thick, gritty fluid. In the center of the tumor, after the fluid was diſcharged, ſhe perceived a ſmall ſoft ſubſtance, of the ſize of a pea, and of a reddiſh colour on the top, which at that time ſhe took for proud fleſh. It gradually increaſed in length and thickneſs, and continued pliable for about three months, when it firſt began to put on an horny appearance. In two years and three months from its firſt formation, made deſperate by the increaſed violence of the pain, ſhe attempted to tear it from her head; and with much difficulty, and many efforts, at length broke it in the middle, and afterwards tore the root from her head, leaving a conſiderable depreſſion which ſtill remains in the part where it grew. Its length altogether, it ſeems, is about five inches, and its circumference at the two ends about one inch; but in the middle rather lefs. It is curled like a ram's horn contorted, and in colour much refembles ifinglafs.

From the lower edge of the depreffion another horn, we are told, is now growing, of the fame colour with the former, in length about three inches, and nearly the thicknefs of a fmall goofe quill; this is lefs contorted than the other, and lies clofe upon the head.

A third horn, fituated about the upper part of the lambdoidal future, is much curved, above an inch in length, and more in circumference at its root: its direction is backwards, with fome elevation from the head. At this place two or three fucceffive horns have been produced, which fhe has conftantly torn away; but, as frefh ones have fpeedily followed, fhe leaves the prefent one unmolefted in hopes of its dropping off.

Befides thefe horny excrefcences, there are two tumors, each of the fize of a large cockle; one upon the upper part, the other about the middle of the left fide of the head; both of them admit of confiderable motion, and feem to contain fluids of unequal confiftence; the upper upper one affording an obſcure fluctuation, the other a very evident one.

The four horns were all preceded by the ſame kind of encyſted tumors, and the fluid in all of them was gritty; the openings from which the matter iſſued were very ſmall, the cyſts collapſed and dried up, leaving the ſubſtance from which the horn proceeded diſtinguiſhable at the bottom. Theſe cyſts gave little pain till the horns began to ſhoot, and then bcame very diſtreſſing, and continued with ſhort intervals till they were removed.

This caſe, we are told, was drawn up by the ſurgeon who attended the patient for many years, and who of courſe had frequent opportunities of ſeeing the diſeaſe in its different ſtages, and acquiring an accurate hiſtory of its ſymptoms.

The ſubject of the ſecond caſe is a middle-aged woman, of the name of Allen, reſident in Leiceſterſhire, and who had an encyſted tumor upon her head, immediately under the ſcalp, very moveable, and evidently containing a fluid. It gave no pain unleſs preſſed upon, and grew to the ſize of a ſmall hen's egg. A few years ago it burſt, and diſcharged a fluid; this diminiſhed in quantity, and in a ſhort time a horny a horny excrefcence, fimilar to thofe above mentioned, grew out from the orifice, which has continued to increafe in fize; and in the month of November 1790, when Mr. Home faw it, was about five inches long, and a little more than an inch in circumference at its bafe. It was a good deal contorted, and its furface was very irregular, having a laminated appearance. It moved readily with the fcalp, and feemed to give no pain upon motion; but, when much handled, the furrounding fkin became inflamed. This woman came to London, and exhibited herfelf as a fhow for money.

That thefe two cafes may not be confidered as peculiar inftances from which no conclufions can be drawn, Mr. Home has thought it right to take notice of fome of the moft remarkable hiftories of this kind that are to be met with in books, and to confider how far they agree with thofe he has ftated, in the general characters that are fufficiently obvious to ftrike a common obferver; for the vague and indefinite terms in which authors exprefs themfelves on this fubject fhow plainly, he thinks, that they did not underftand the nature of the difeafe, and their accounts of it, he obferves, are not very fatiffactory to their readers.

In

In the *Ephemerides Academiæ Naturæ Curiosorum* he has found two cafes of horns growing from the human body. One of thefe inftances was a German woman *, who had feveral fwellings, or ganglions, upon different parts of her head, from one of which a horn grew. The other was a nobleman †, who had a fmall tumor, about the fize of a nut, growing upon the parts covering the two laft or lowermoft vertebræ of the back. It continued for ten years, without undergoing any apparent change; but afterwards enlarged in fize, and a horny excrefcence grew out from it.

In the Hiftory of the Royal Medical Society at Paris ‡, he has met with an account of a woman, 97 years old, who had feveral tumors on her head, which had been 14 years in growing to the ftate they were in at that time: fhe had alfo a horn which had originated from a fimilar tumor. The horn was very moveable, being attached to the fcalp, without any adhe-

* Ephem. Acad. Nat. Cur. Dec. iii. An. V. Append. p. 148.

† Ibid. Dec. i. An. I. Obfervat. 30.

‡ Hiftoire de la Société Royale de Médecine, 1776, p. 316.

ſion to the ſcull. It was ſawn off, but grew again, and although the operation was repeated ſeveral times, the horn always returned.

Bartholine *, he obſerves, takes notice of a woman who had a tumour under the ſcalp, covering the temporal muſcle. This gradually enlarged, and a horn grew from it, which had become twelve inches long in the year 1646, when it was ſeen by Bartholine, who has given a repreſentation of it, which, Mr. Home tells us, bears a very accurate reſemblance to that which he has mentioned to have ſeen in November, 1790. No tumour or ſwelling is expreſſed in the figure; but the horn is coming directly out from the ſurface of the ſkin.

Mr. Home's next reference is to Leigh's Natural Hiſtory of Cheſhire, where a woman is mentioned to have lived in the year 1668, who had a tumor or wen upon her head for thirty-two years, which afterwards enlarged, and two horns grew out of it; ſhe was then ſeventy-two years old.

There is a horny excreſcence in the Britiſh Muſeum, which is eleven inches long, and two

* *Vide* Thomæ Bartholini Hiſtor. anat. rar. cent. 1. hiſt. 78.

inches and a half in circumference at the baſe, or thickeſt part. The following account of this horn, taken from the records of the Muſeum, was given to our author by Dr. Gray. " A woman, " named French, who lived near Tenterden, " had a tumour or wen upon her head, which " increaſed to the ſize of a walnut; and in the " forty-eighth year of her age this horn began " to grow, and in four years arrived at its pre- " ſent ſize *."

There are many ſimilar hiſtories of theſe horny excreſcences, our author obſerves, in the books he has quoted, and in ſeveral others; but thoſe mentioned above are the moſt accurate and particular with reſpect to their growth, and in all of them we find the origin was from a tumour,

* In a note to this part of his paper Mr. Home gives the following extract from the Minutes of the Royal Society, Feb. 14, 1704-5.

" A Letter was read from Dr. Chariere, at Barnſtaple, " concerning a horn, ſeven inches long, cut off the ſecond " vertebra of the neck of a woman in that neighbour- " hood.

" Dr. Gregory ſaid, that one of ſeven inches long, and " of a dark brown colour, was cut off from a woman's " temple at Edinburgh.

" Dr. Norris ſaid, that two horns had been cut off from a woman's head in Cheſhire."

as as in the two cafes he has related; and although the nature of the tumour is not particularly mentioned, there can be no doubt, he thinks, of its having been of the encyfted kind, fince in its progrefs it exactly refembled them, remaining ftationary for a long time, and then coming forwards to the fkin; and the horn being much fmaller than the tumour previoufly to the formation of the horn, is a proof, he obferves, that the tumour muft have burft, and difcharged its contents.

From the foregoing account it will, he thinks, appear evident, that thefe horny excrefcences are not to be ranked among the appearances called *lufus naturæ*: nor are they, in his opinion, altogether the product of difeafe, although undoubtedly the confequence of a local difeafe which has previoufly exifted; they are, he contends, more properly fpeaking, the refult of certain operations in the part for its own reftoration; but the actions of the animal œconomy being unable to bring them back to their original ftate, this fpecies of excrefcence is formed as a fubftitute for the natural cuticular covering.

To explain the manner in which thefe horns are formed, he has thought it neceffary to confider the nature of encyfted tumours a little more fully; fully; and in doing ſo he makes it appear, that this particular ſpecies does not differ in its principle, nor materially in its effects, from many others which are not uncommonly met with in the human body, as well as in thoſe of many other animals, which, as they are more frequent in their occurrence, are alſo much better underſtood.

Encyſted tumours, he obſerves, differ exceedingly among themſelves, both in the nature of their contents, and in their progreſs towards the external ſurface of the body. Many of them have no reference to our preſent purpoſe: it is only the more indolent kind to which he means now to advert; ſome of theſe, he remarks, when examined, are not found to contain a fluid, but a ſmall quantity of thick, curd-like matter, mixed with cuticle broken down into ſmall parts, and, upon expoſing the internal ſurface of the cyſt, it is found to have an uniform cuticular covering adhering to it, ſimilar to that of the cutis on the ſurface of the body, from which it only differs in being thinner, and more delicate, bearing a greater reſemblance to that which covers the lips. Others of this kind, inſtead of having cuticle for their contents, are filled with hair mixed with a curdled ſubſtance, or hair without without any admixture whatever, and have a fimilar kind of hair growing upon their internal furface, which is likewife covered with a cuticle. Thefe cuticular encyfted tumours were, he believes, firft accurately examined by Mr. Hunter, to whom we are likewife indebted for an explanation of the mode in which the parts acquire this particular ftructure.

Mr. Hunter, it feems, confiders the internal furface of the cyft to be fo circumftanced refpecting the body as to lofe the ftimulus of being an internal part, and receive the fame impreffion from its contents, either from their nature, or the length of application, as the furface of the fkin does from its external fituation. It, therefore, takes on actions fuited to fuch ftimuli, undergoes a change in its ftructure, and acquires a difpofition fimilar to the cutis, and is confequently poffeffed of the power of producing cuticle and hair. What the mode of action is, by which this change is brought about, is not eafily determined; but from the indolence of thefe complaints, it moft probably requires a confiderable length of time to produce it. That the lining of the cyft really does poffefs powers fimilar to cutis, is proved, our author thinks, by the following circumftances:—that it has a power of forming a fucceffion of cuticles like the common fkin ; and what is thrown off in this way is found in the cavity of the cyft. It has a fimilar power, he obferves, refpecting hair, and fometimes the cavity is filled with it, fo great a quantity has been fhed by the internal furface. Befides thefe circumftances, the hair found in the cyft correfponds in appearance with that which grows upon the body of the animal; and when encyfted tumours of this kind form in fheep, they contain wool. What is ftill more curious, when fuch cyfts are laid open, the internal furface undergoes no change from expofure, the cut edges cicatrife, and the bottom of the bag remains ever after an external furface. Different fpecimens, we are told, illuftrative of the above-mentioned circumftances, are preferved in Mr. Hunter's collection of difeafes.

The cyfts that produce horny excrefcences (which are only another modification of cuticle) are, in our author's opinion, very improperly confidered as giving rife to horns; for if we examine the mode in which this fubftance grows, we fhall find it, he obferves, the fame with the human nails, coming directly out from the furface of the cutis. It differs from the nails nails in not being fet upon the fkin by a thin edge, but by a furface of fome breadth, with a hollow in the middle, exactly in the fame manner as the horn of the rhinoceros *; at leaft this, he affures us, is evidently the cafe in the fpecimen preferved in the Britifh Mufeum, and in one which grew out from the tip of a fheep's ear; they are alfo, he adds, folid, or nearly fo, in their fubftance.

This mode of growth, our author obferves, is very different from that of horns, which are all formed upon a core, either of bone or foft parts, by which means they have a cavity in them, a ftructure peculiar to this kind of cuticular fubftance.

Encyfted tumours in different animals would appear, he thinks, from thefe obfervations, to be confined in their production to the cuticular fubftance proper to the animal in which they take place; for, although cuticle, hair, nail, hoof, and horn, are equally productions of ani-

* "The horn of the rhinoceros is a cuticular appen- "dage to the fkin, fimilar to nails and other cuticular ex- "crefcences, being in no refpect allied to horns but in the "external appearance."—*Note by Mr. Home.*

mal ſubſtance, only differing in trivial circumſtances from each other, we do not, he remarks, find in the human ſubject any inſtance of an encyſted tumour containing a ſubſtance different from the cuticle, hair, and nails of the human body, to which laſt the horny excreſcences, the ſubject of the preſent paper, are, according to our author's obſervations, very cloſely allied, both in growth, ſtructure, and external appearance; and when of ſome length, they are found, we are told, to be ſo brittle as to break in two, upon being roughly handled, which could not happen either to hoof or horn. In the ſheep, he obſerves, they produce wool inſtead of hair; and in one inſtance in that animal, where they gave riſe to an horny excreſcence, he found it leſs compact in its texture, and leſs brittle than ſimilar appearances in the human ſubject; upon being divided longitudinally, the cut ſurface had more the appearance of hoof, and was more varied in colour, than nail.

Encyſted tumours being capable of producing horns, upon the principle here laid down, is contrary, Mr. Home obſerves, to the uſual operations of nature; for horns are not a production duction from the cutis, and although not always formed upon a bony core, but frequently upon a soft pulp, that substance differs from common cutis in its appearance, and extends a considerable way into the horn: it is, therefore, he thinks, probable, that this pulp requires a particular process for its formation. In support of this opinion he gives the following fact:

" A sheep, about four years old, had a large

" horn, three feet long, growing upon its flank.

" It had no connection with bone, and appear-

" ed to be only attached to the external skin.

" It dropped off in consequence of its weight

" having produced ulceration in the soft parts

" to which it adhered. Upon examining it

" there was a fleshy substance, seven inches long,

" of a fibrous texture, filling up its cavity upon

" which the horn had been formed."

Towards the conclusion of his paper Mr. Home observes, that the cases of horns, as they are commonly termed, upon the human head, are no more than cuticular productions arising from a cyst, which in its nature is a variety of those tumours described by Mr. Hunter under the general name of cuticular encysted tumours.

The principle upon which the production of these excrescences depends being once explained, the modes of preventing their formation, and removing them when formed, will, he thinks, be readily underftood, the deftruction of the cyft being all that is required for that purpofe. This, he obferves, may be done before the tumour opens externally, or even after the excrefcence has begun to fhoot out, and will be better effected by diffection than efcharotics, fince the fuccefs of the operation depends upon the whole of the bag being removed.

Thefe encyfted tumours, he obferves, when confidered as varieties of the fame difeafe, form a very complete and beautiful feries of the different modes by which the powers of the animal œconomy produce a fubftitute for the common cuticle upon parts which have been fo much affected by difeafe as to be unable to reftore themfelves to a natural ftate.

